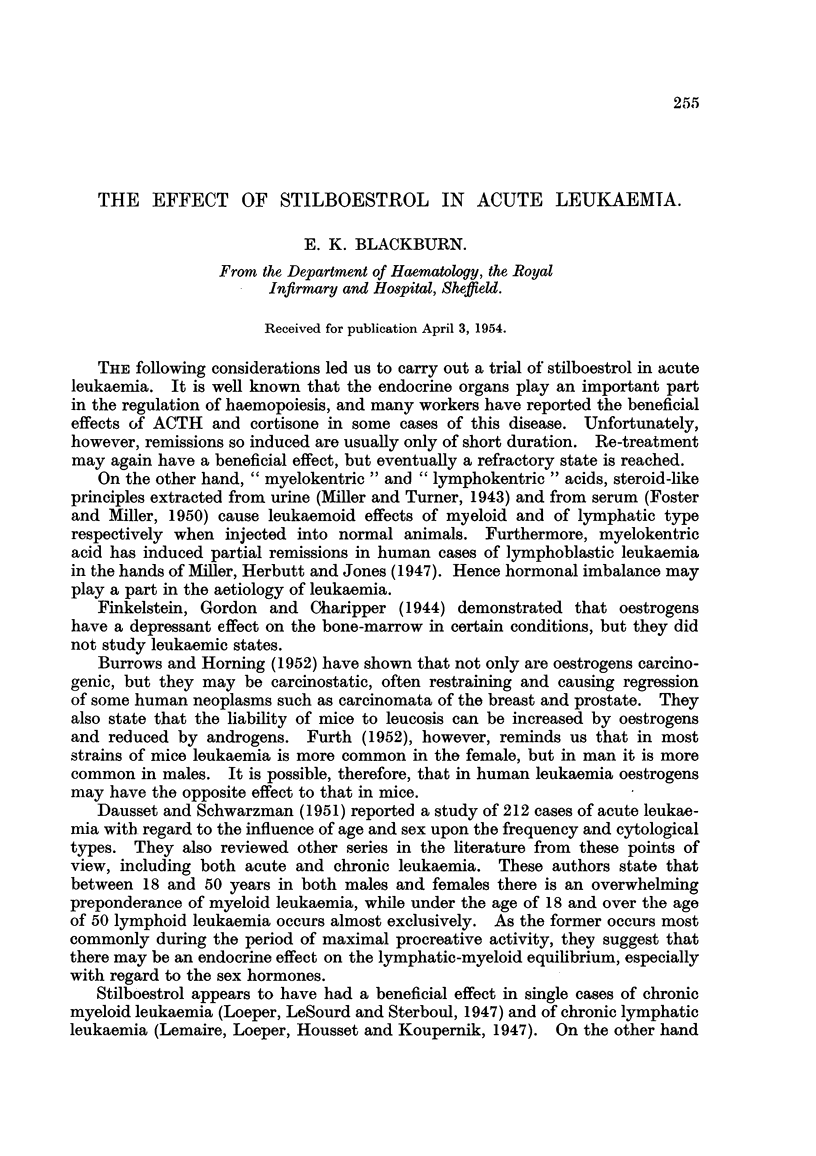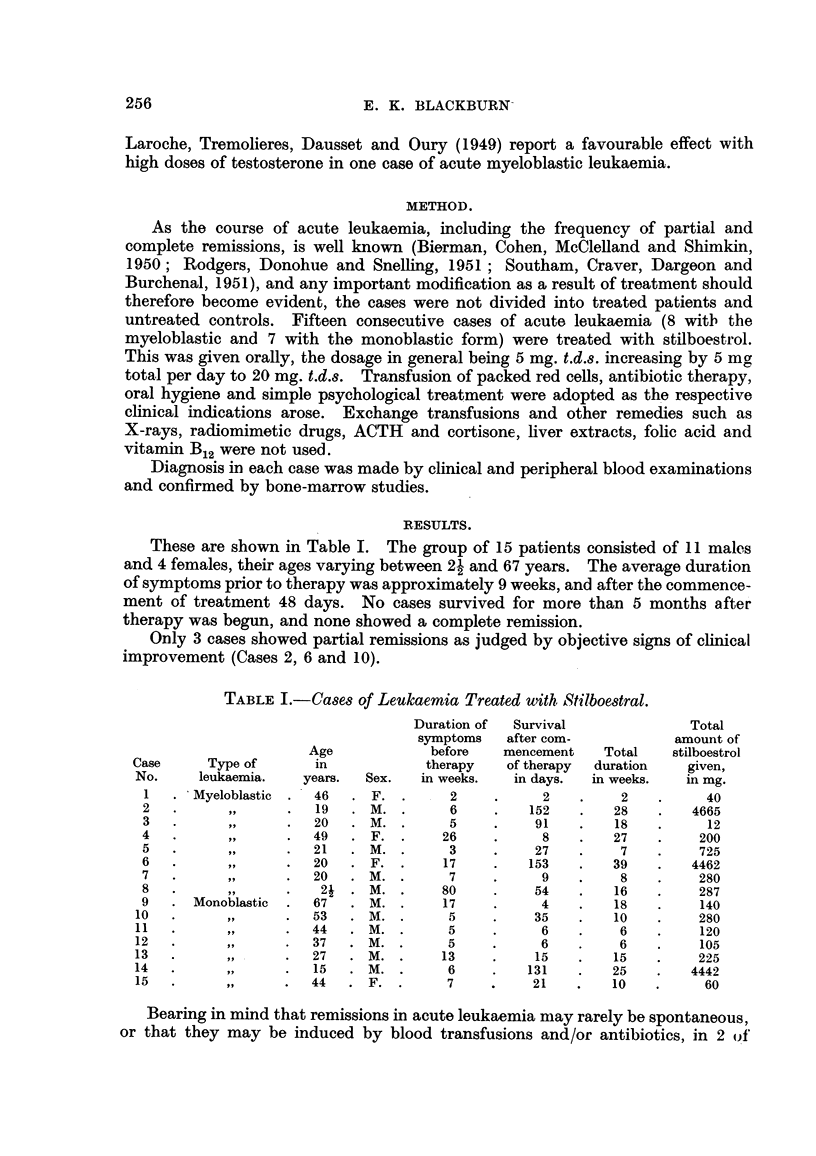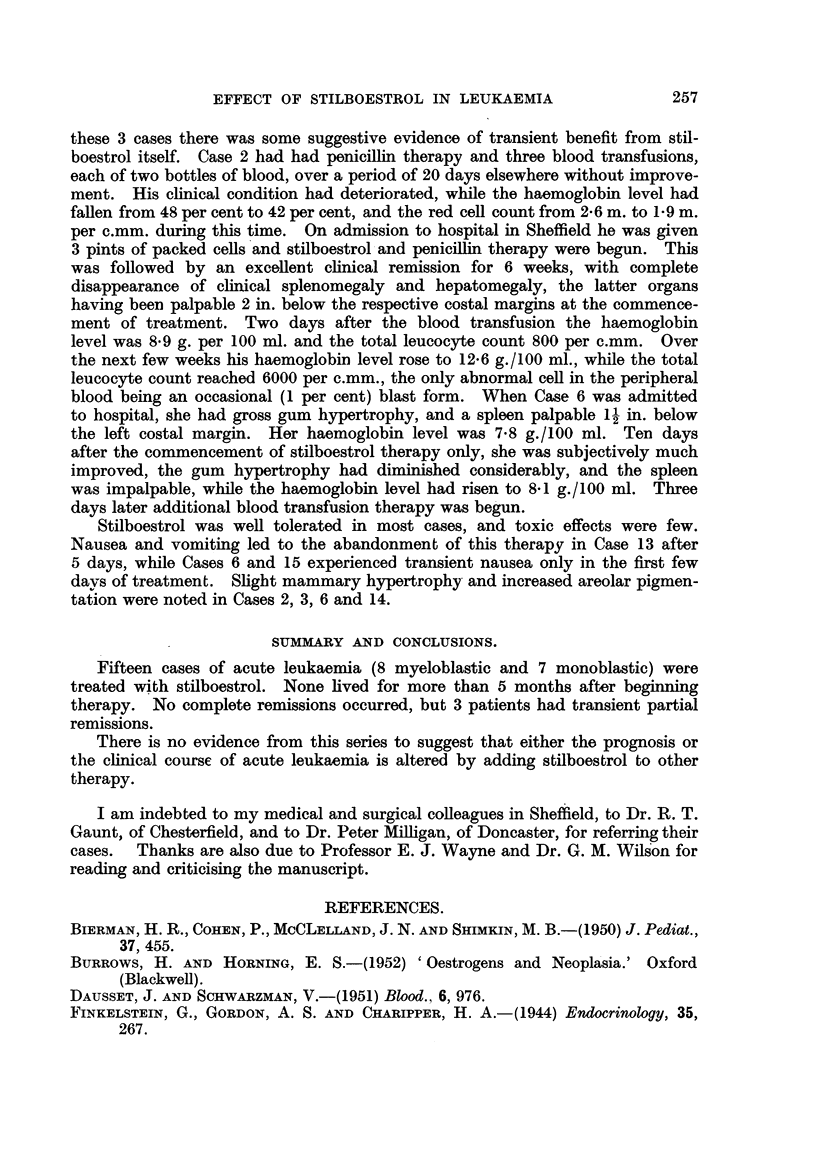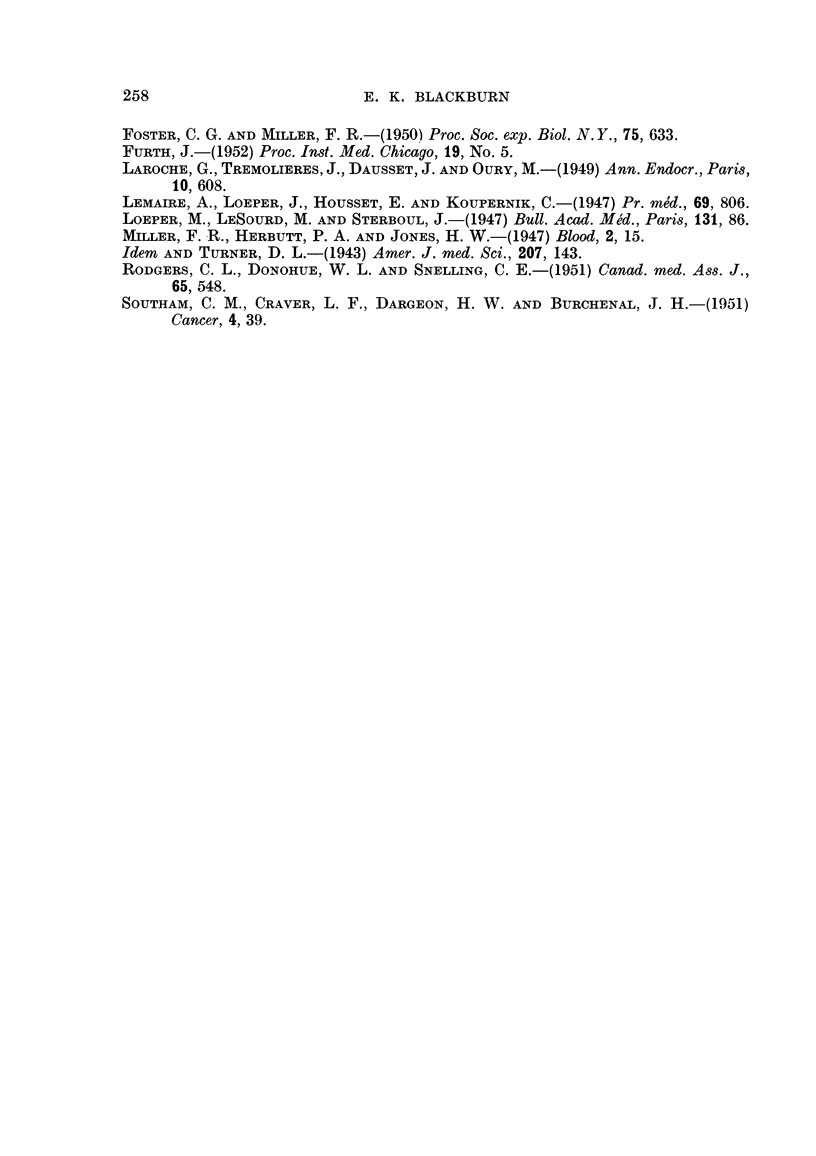# The Effect of Stilboestrol in Acute Leukaemia

**DOI:** 10.1038/bjc.1954.26

**Published:** 1954-06

**Authors:** E. K. Blackburn


### THE EFFECT OF STILBOESTROL IN ACUTE LEUKAEMIA.

E. K. BLACKBURN.

*From the Department of Haematology, the Royal Infirmary and Hospital, Sheffield.*

Received for publication April 3, 1954.

THE following considerations led us to carry out a trial of stilboestrol in acute leukaemia. It is well known that the endocrine organs play an important part in the regulation of haemopoiesis, and many workers have reported the beneficial effects of ACTH and cortisone in some cases of this disease. Unfortunately, however, remissions so induced are usually only of short duration. Re-treatment may again have a beneficial effect, but eventually a refractory state is reached.

On the other hand, " myelokentric " and " lymphokentric " acids, steroid-like principles extracted from urine (Miller and Turner, 1943) and from serum (Foster and Miller, 1950) cause leukaemoid effects of myeloid and of lymphatic type respectively when injected into normal animals. Furthermore, myelokentric acid has induced partial remissions in human cases of lymphoblastic leukaemia in the hands of Miller, Herbutt and Jones (1947). Hence hormonal imbalance may play a part in the aetiology of leukaemia.

Finkelstein, Gordon and Charipper (1944) demonstrated that oestrogens have a depressant effect on the bone-marrow in certain conditions, but they did not study leukaemic states.

Burrows and Horning (1952) have shown that not only are oestrogens carcinogenic, but they may be carcinostatic, often restraining and causing regression of some human neoplasms such as carcinomata of the breast and prostate. They also state that the liability of mice to leucosis can be increased by oestrogens and reduced by androgens. Furth (1952), however, reminds us that in most strains of mice leukaemia is more common in the female, but in man it is more common in males. It is possible, therefore, that in human leukaemia oestrogens may have the opposite effect to that in mice.

Dausset and Schwarzman (1951) reported a study of 212 cases of acute leukaemia with regard to the influence of age and sex upon the frequency and cytological types. They also reviewed other series in the literature from these points of view, including both acute and chronic leukaemia. These authors state that between 18 and 50 years in both males and females there is an overwhelming preponderance of myeloid leukaemia, while under the age of 18 and over the age of 50 lymphoid leukaemia occurs almost exclusively. As the former occurs most commonly during the period of maximal procreative activity, they suggest that there may be an endocrine effect on the lymphatic-myeloid equilibrium, especially with regard to the sex hormones.

Stilboestrol appears to have had a beneficial effect in single cases of chronic myeloid leukaemia (Loeper, LeSourd and Sterboul, 1947) and of chronic lymphatic leukaemia (Lemaire, Loeper, Housset and Koupernik, 1947). On the other hand

Laroche, Tremolieres, Dausset and Oury (1949) report a favourable effect with high doses of testosterone in one case of acute myeloblastic leukaemia.

### METHOD.

As the course of acute leukaemia, including the frequency of partial and complete remissions, is well known (Bierman, Cohen, McClelland and Shimkin, 1950 ; Rodgers, Donohue and Snelling, 1951 ; Southam, Craver, Dargeon and Burchenal, 1951), and any important modification as a result of treatment should therefore become evident, the cases were not divided into treated patients and untreated controls. Fifteen consecutive cases of acute leukaemia (8 with the myeloblastic and 7 with the monoblastic form) were treated with stilboestrol. This was given orally, the dosage in general being 5 mg. *t.d.s.* increasing by 5 mg total per day to 20 mg. *t.d.s.* Transfusion of packed red cells, antibiotic therapy, oral hygiene and simple psychological treatment were adopted as the respective clinical indications arose. Exchange transfusions and other remedies such as X-rays, radiomimetic drugs, ACTH and cortisone, liver extracts, folic acid and vitamin $B_{12}$ were not used.

Diagnosis in each case was made by clinical and peripheral blood examinations and confirmed by bone-marrow studies.

### RESULTS.

These are shown in Table I. The group of 15 patients consisted of 11 males and 4 females, their ages varying between 2½ and 67 years. The average duration of symptoms prior to therapy was approximately 9 weeks, and after the commencement of treatment 48 days. No cases survived for more than 5 months after therapy was begun, and none showed a complete remission.

Only 3 cases showed partial remissions as judged by objective signs of clinical improvement (Cases 2, 6 and 10).

TABLE I.—*Cases of Leukaemia Treated with Stilboestral.*

| Case No. | Type of leukaemia. | Age in years. | Sex. | Duration of symptoms before therapy in weeks. | Survival after commencement of therapy in days. | Total duration in weeks. | Total amount of stilboestrol given, in mg. |
|---|---|---|---|---|---|---|---|
| 1 | Myeloblastic | 46 | F. | 2 | 2 | 2 | 40 |
| 2 | ,, | 19 | M. | 6 | 152 | 28 | 4665 |
| 3 | ,, | 20 | M. | 5 | 91 | 18 | 12 |
| 4 | ,, | 49 | F. | 26 | 8 | 27 | 200 |
| 5 | ,, | 21 | M. | 3 | 27 | 7 | 725 |
| 6 | ,, | 20 | F. | 17 | 153 | 39 | 4462 |
| 7 | ,, | 20 | M. | 7 | 9 | 8 | 280 |
| 8 | ,, | 2½ | M. | 80 | 54 | 16 | 287 |
| 9 | Monoblastic | 67 | M. | 17 | 4 | 18 | 140 |
| 10 | ,, | 53 | M. | 5 | 35 | 10 | 280 |
| 11 | ,, | 44 | M. | 5 | 6 | 6 | 120 |
| 12 | ,, | 37 | M. | 5 | 6 | 6 | 105 |
| 13 | ,, | 27 | M. | 13 | 15 | 15 | 225 |
| 14 | ,, | 15 | M. | 6 | 131 | 25 | 4442 |
| 15 | ,, | 44 | F. | 7 | 21 | 10 | 60 |

Bearing in mind that remissions in acute leukaemia may rarely be spontaneous, or that they may be induced by blood transfusions and/or antibiotics, in 2 of these 3 cases there was some suggestive evidence of transient benefit from stilboestrol itself. Case 2 had had penicillin therapy and three blood transfusions, each of two bottles of blood, over a period of 20 days elsewhere without improvement. His clinical condition had deteriorated, while the haemoglobin level had fallen from 48 per cent to 42 per cent, and the red cell count from 2·6 m. to 1·9 m. per c.mm. during this time. On admission to hospital in Sheffield he was given 3 pints of packed cells and stilboestrol and penicillin therapy were begun. This was followed by an excellent clinical remission for 6 weeks, with complete disappearance of clinical splenomegaly and hepatomegaly, the latter organs having been palpable 2 in. below the respective costal margins at the commencement of treatment. Two days after the blood transfusion the haemoglobin level was 8·9 g. per 100 ml. and the total leucocyte count 800 per c.mm. Over the next few weeks his haemoglobin level rose to 12·6 g./100 ml., while the total leucocyte count reached 6000 per c.mm., the only abnormal cell in the peripheral blood being an occasional (1 per cent) blast form. When Case 6 was admitted to hospital, she had gross gum hypertrophy, and a spleen palpable 1½ in. below the left costal margin. Her haemoglobin level was 7·8 g./100 ml. Ten days after the commencement of stilboestrol therapy only, she was subjectively much improved, the gum hypertrophy had diminished considerably, and the spleen was impalpable, while the haemoglobin level had risen to 8·1 g./100 ml. Three days later additional blood transfusion therapy was begun.

Stilboestrol was well tolerated in most cases, and toxic effects were few. Nausea and vomiting led to the abandonment of this therapy in Case 13 after 5 days, while Cases 6 and 15 experienced transient nausea only in the first few days of treatment. Slight mammary hypertrophy and increased areolar pigmentation were noted in Cases 2, 3, 6 and 14.

### SUMMARY AND CONCLUSIONS.

Fifteen cases of acute leukaemia (8 myeloblastic and 7 monoblastic) were treated with stilboestrol. None lived for more than 5 months after beginning therapy. No complete remissions occurred, but 3 patients had transient partial remissions.

There is no evidence from this series to suggest that either the prognosis or the clinical course of acute leukaemia is altered by adding stilboestrol to other therapy.

I am indebted to my medical and surgical colleagues in Sheffield, to Dr. R. T. Gaunt, of Chesterfield, and to Dr. Peter Milligan, of Doncaster, for referring their cases. Thanks are also due to Professor E. J. Wayne and Dr. G. M. Wilson for reading and criticising the manuscript.

### REFERENCES.

BIERMAN, H. R., COHEN, P., McCLELLAND, J. N. AND SHIMKIN, M. B.—(1950) *J. Pediat.*, **37**, 455.

BURROWS, H. AND HORNING, E. S.—(1952) 'Oestrogens and Neoplasia.' Oxford (Blackwell).

DAUSSET, J. AND SCHWARZMAN, V.—(1951) *Blood.*, **6**, 976.

FINKELSTEIN, G., GORDON, A. S. AND CHARIPPER, H. A.—(1944) *Endocrinology*, **35**, 267.

Foster, C. G. and Miller, F. R.—(1950) *Proc. Soc. exp. Biol. N.Y.*, **75**, 633.
Furth, J.—(1952) *Proc. Inst. Med. Chicago*, **19**, No. 5.
Laroche, G., Tremolieres, J., Dausset, J. and Oury, M.—(1949) *Ann. Endocr., Paris*, **10**, 608.
Lemaire, A., Loeper, J., Housset, E. and Koupernik, C.—(1947) *Pr. méd.*, **69**, 806.
Loeper, M., LeSourd, M. and Sterboul, J.—(1947) *Bull. Acad. Méd., Paris*, **131**, 86.
Miller, F. R., Herbutt, P. A. and Jones, H. W.—(1947) *Blood*, **2**, 15.
*Idem* and Turner, D. L.—(1943) *Amer. J. med. Sci.*, **207**, 143.
Rodgers, C. L., Donohue, W. L. and Snelling, C. E.—(1951) *Canad. med. Ass. J.*, **65**, 548.
Southam, C. M., Craver, L. F., Dargeon, H. W. and Burchenal, J. H.—(1951) *Cancer*, **4**, 39.